# News sensitive stock market prediction: literature review and suggestions

**DOI:** 10.7717/peerj-cs.490

**Published:** 2021-05-04

**Authors:** Shazia Usmani, Jawwad A. Shamsi

**Affiliations:** Systems Research Laboratory, FAST-National University of Computer and Emerging Sciences, Karachi, Pakistan

**Keywords:** Stock prediction, Text mining, Feature extraction, Event extraction, NLP, Machine learning, Neural network, Sentiment analysis

## Abstract

Stock market prediction is a challenging task as it requires deep insights for extraction of news events, analysis of historic data, and impact of news events on stock price trends. The challenge is further exacerbated due to the high volatility of stock price trends. However, a detailed overview that discusses the overall context of stock prediction is elusive in literature. To address this research gap, this paper presents a detailed survey. All key terms and phases of generic stock prediction methodology along with challenges, are described. A detailed literature review that covers data preprocessing techniques, feature extraction techniques, prediction techniques, and future directions is presented for news sensitive stock prediction. This work investigates the significance of using structured text features rather than unstructured and shallow text features. It also discusses the use of opinion extraction techniques. In addition, it emphasizes the use of domain knowledge with both approaches of textual feature extraction. Furthermore, it highlights the significance of deep neural network based prediction techniques to capture the hidden relationship between textual and numerical data. This survey is significant and novel as it elaborates a comprehensive framework for stock market prediction and highlights the strengths and weaknesses of existing approaches. It presents a wide range of open issues and research directions that are beneficial for the research community.

## Introduction

Stock market trends are extremely volatile in nature that makes prediction quite hard. This volatile nature attracts researchers to investigate sophisticated techniques for better prediction. Prediction of stock market trends with high accuracy generates significant revenue. Fundamental and technical analyses are two basic approaches used for stock trend prediction. Technical analysis inspects past data and volumes of stock prices while fundamental analysis not only considers stock statistics but also evaluates industry’s performance, political events, and economic circumstances (Patel et al., 2015; Milosevic, 2016). Fundamental analysis is more realistic because it evaluates the market in a broader scope. This survey puts emphasis on research work based on fundamental analysis, where textual data is considered along with stock price historical data for stock trend prediction.

There are many sources of textual data like news, tweets, and annual reports, etc. which could be analyzed to mine significant information. Textual data, especially news, is a better source of hidden information than numeric data because it permits to predict financial trends with its justification (Chan & Franklin, 2011). For instance, a news article on a company with words or phrases like “resignation”, “risk of default” helps the investor to predict a decrease in the company’s stock prices. Furthermore, news about many uncertain factors can affect stock market trends (Nassirtoussi et al., 2015). For instance, economic and political shocks, war, civil unrest, terrorism, and natural disasters etc. Therefore, there is a great need for better knowledge discovery mechanisms from textual data.

Feature extraction is a fundamental step in prediction where input data is reduced into more manageable form for further processing. Most of the previous work on news sensitive stock trend prediction adopted shallow features extraction techniques which are unstructured and where words are represented as features. For instance, Bag-of-Words (BoW), noun phrases, and named entities (Schumaker & Chen, 2009). This is contrary to the structured feature extraction technique where a combination of words, nouns, and verbs are used. Unlike structured feature extraction techniques, shallow feature extraction techniques are not able to capture a complete event in the form of structured entity-relation information. Consequently, shallow features make it complicated to represent the impact of news events on stock market trend prediction (Ding et al., 2014).

Events extracted from news articles may play a significant role in stock market trend prediction. Sophisticated Natural Language Processing (NLP) technologies enable more accurate structured representation of events than shallow features. But structured representation of events increases sparsity, which most probably decreases the predictive power (Ding et al., 2015). This issue is solved by using event embedding, which are dense vectors. Event embedding is used to reduce sparsity due to structured representation of events by comprising syntactically or semantically similar events into similar vectors. But event embedding suffers from some limitations due to the lack of background knowledge (Ding et al., 2016). For instance, two events with similar words may have similar embedding. However, they do not have any causal or logical relation. Integrating knowledge base in learning event embedding will result in better event embedding.

Shallow features and event based features represent facts about text while expressing subjective textual information is another way for text analysis. Sentiment analysis is a widely used approach to infer emotion from textual data that represents subjective information. It is a major area of interest in today’s text analytics. In news sensitive stock prediction, it is used to extract news polarity using machine learning and sentiment dictionary based approaches (Li, Wu & Wang, 2020).

Features extracted from input are fed into machine learning algorithm for prediction. In existing literature, two types of machine learning techniques are used for stock trend prediction. Shallow learning is a machine learning technique, where composition layers are few like Support Vector Machine (SVM) and Artificial Neural Network (ANN). While deep learning technique contains many hidden layers like Convolutional Neural Network (CNN). The elegance of deep learning is to extract features and learn classification (Pasupa & Sunhem, 2016; Vargas, De Lima & Evsukoff, 2017; Dutta, 2018; Long, Lu & Cui, 2019).

Considering the impact and potential of news on stock market performance, there is a significant need to analyze, assess, and evaluate techniques of news sensitive stock market prediction. This paper is motivated to address this need. In the context of news sensitive stock trend prediction, we have three questions to direct this research work: (i) What is the generic methodology to perform prediction? (ii) Which approaches have been used for text processing and how these existing approaches can be improved? (iii) Which machine learning algorithms have more potential to model the selected domain?

Based on the above research questions, an extensive literature review related to stock prediction is discussed and guidelines are suggested to forecast the influence of news events on the stock market. The main attainments of this paper are as follows:

1. A thorough research review for stock trend prediction in three areas:
  - Study of stock trend prediction based on financial time series data and preprocessing of financial data.
  - Research based on text preprocessing and feature extraction techniques.
  - Investigation about prediction algorithms to analyze the influence of textual and numerical features on the stock market.
2. Based on reviewed literature, solutions are identified that guide to resolve challenges found in the generic phases of a stock prediction task.
3. Discussion about open issues and research directions, which can be further investigated and explored by the community.

**Figure 1:**
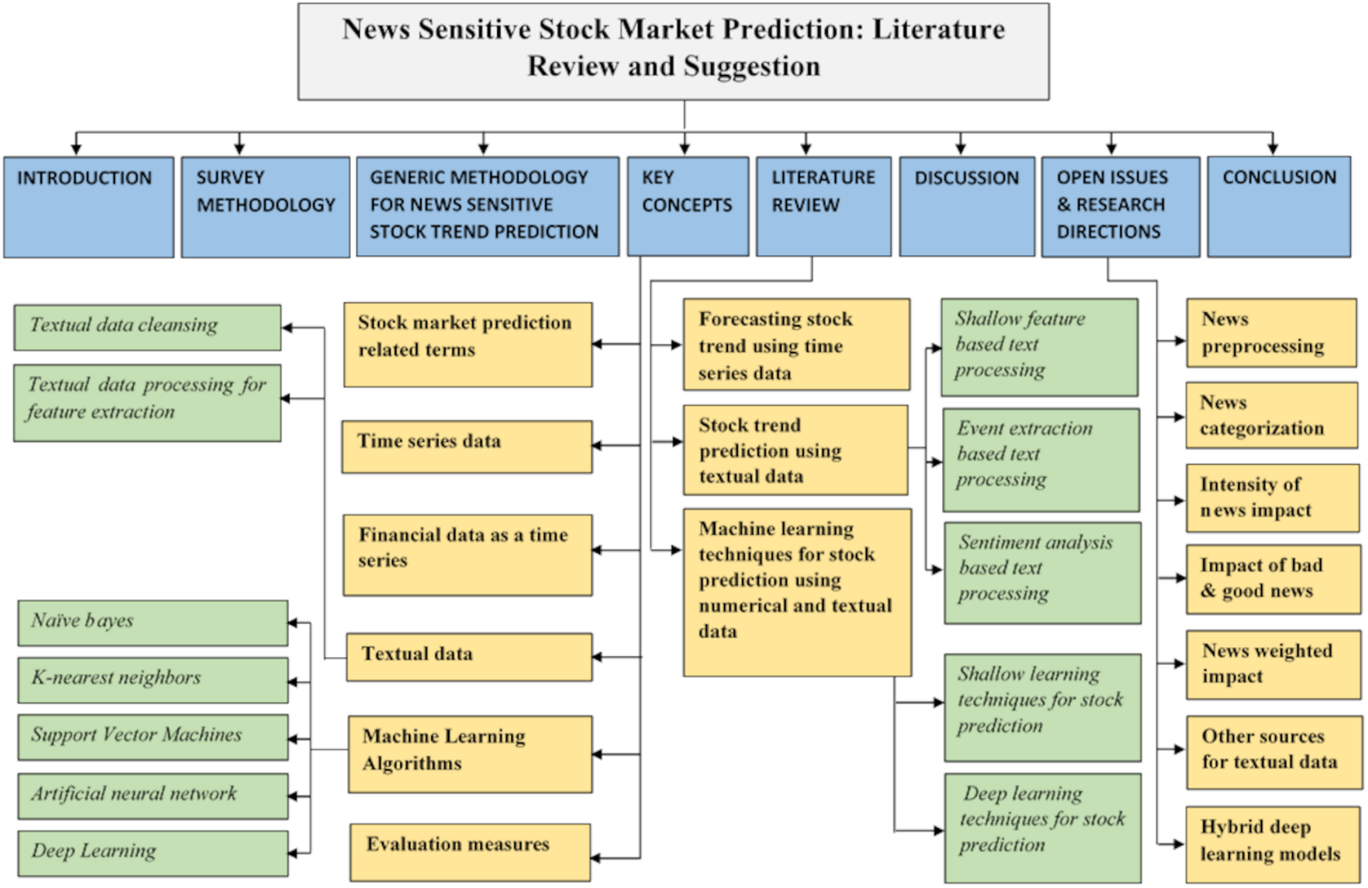
Organization of the survey.

This survey is useful in understanding the needs, fundamentals, frameworks, and techniques for stock market trend analysis. This research work is organized in eight sections. Survey organization is shown in Fig. 1 and survey methodology is discussed in “Survey Methodology”. Generic methodology is discussed along with main challenges in “Generic Methodology for News Sensitive Stock Trend Prediction”. In “Key Concepts”, important terminologies and key concepts are discussed. Literature review is presented in “Literature review”. Furthermore, in “Discussion”, discussion on the reviewed literature techniques is presented along with opportunities suggested to tackle challenges identified in “Generic Methodology for News Sensitive Stock Trend Prediction”. “Open Issues and Research Directions” discusses the open issues and research directions and “Conclusions” concludes this review.

## Survey methodology

This survey is based on research articles from leading journals and conferences. The main research questions that lead this survey: (i) What are the machine learning methods proposed in literature for stock prediction using financial and textual data? (ii) What are the techniques proposed in literature for preprocessing and feature extraction of financial and textual data? (iii) What are the key challenges, opportunities, and open issues in this domain?

“Stock prediction”, categorically is the seed key term for searching relevant literature in this research. Moreover, other search terms are combined using Boolean operator AND with seed term in order to make the searching criteria more specific. This research study emphasizes the domain of stock prediction using machine learning so the other relevant terms are “machine learning”, “artificial intelligence”, “artificial neural network”, “deep learning”, “stock price”, “news headlines”, “event extraction”, “text mining”, “sentiment analysis”, “sentiment lexicon”, and “time series analysis”.

**Figure 2:**
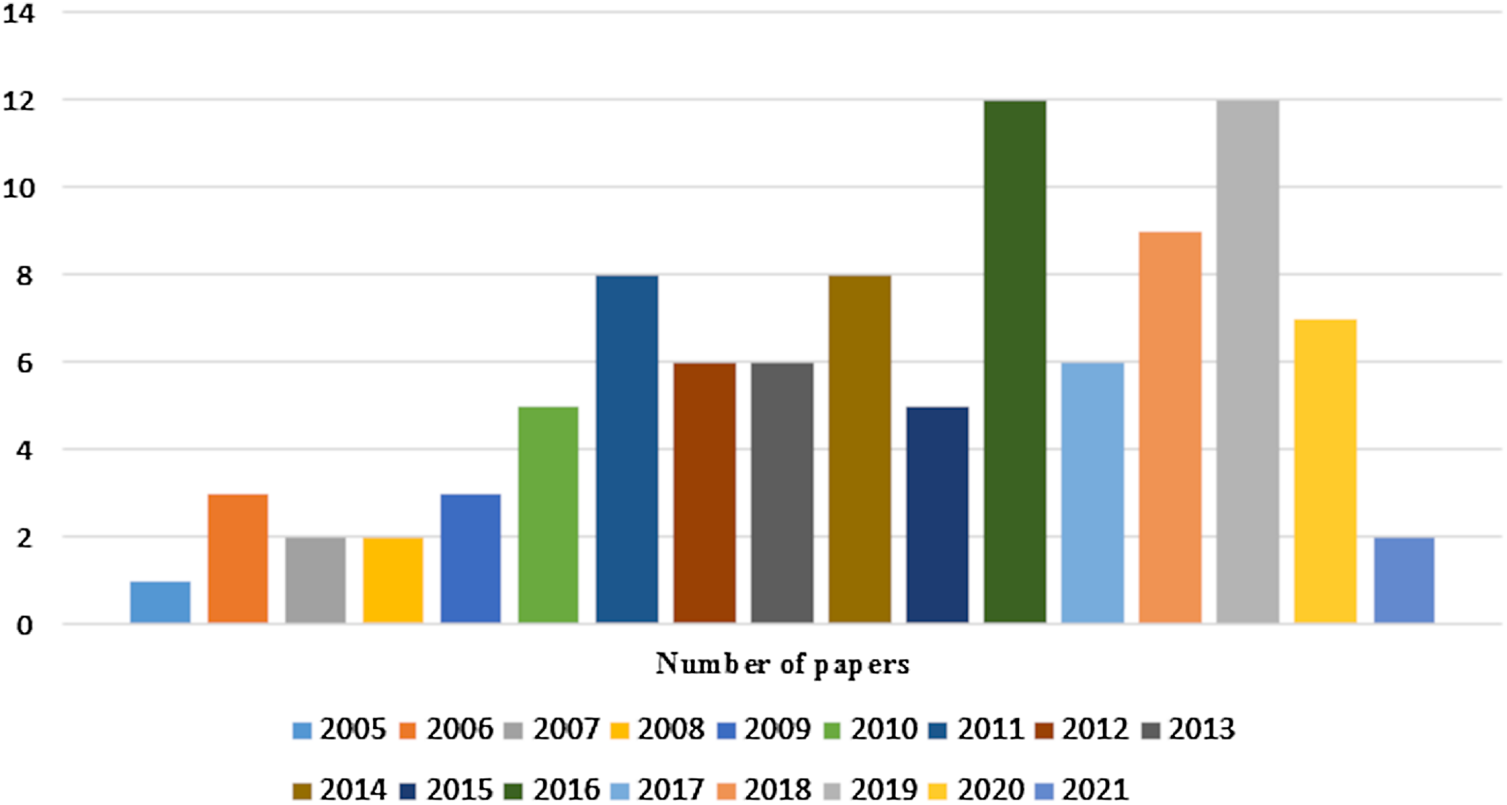
Number of research papers selected per year.

Initially more than 100 papers are selected but not all are included. The main criterion to exclude some of the research papers are their research objective, which do not come in the scope of this study. To cover the domain background as well as state of the art techniques in a wide range, papers were selected from the year 1999 to 2021. Finally, 99 papers are included in this survey. Chart in Fig. 2 shows the number of selected research papers per year.

## Generic methodology for news sensitive stock trend prediction

**Figure 3:**
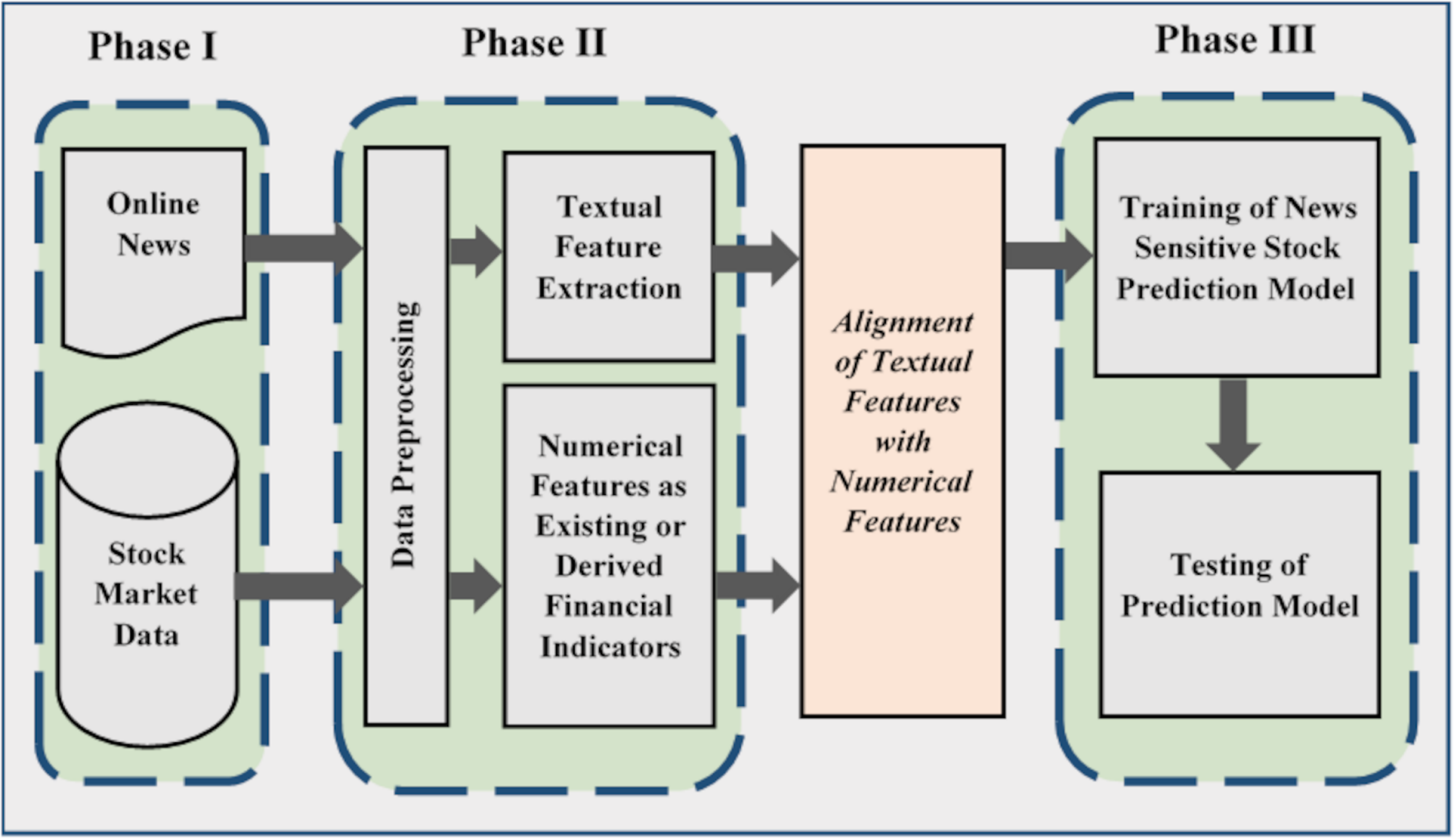
Generic methodology for news sensitive stock trend prediction.

Figure 3 shows the stock prediction generic methodology along with its main phases. The presented generic methodology is adapted from Nassirtoussi et al. (2015) and Cavalcante et al. (2016). It is divided into three phases where the first phase performs data collection. Stock market data can be downloaded from their official websites. Similarly, online news can be downloaded or scrapped from relevant websites. This data can be stored in different file formats for further processing. For example, a CSV (“comma-separated values”) is a simple file format used to store tabular data.

Second phase performs data preprocessing and feature extraction. In the context of news sensitive stock prediction, numerical and textual data is processed separately. Market data is preprocessed to remove inconsistencies and noise to achieve better prediction accuracy. It is also processed in order to select features and derive new features from existing one to align with textual features (Chen et al., 2019; Picasso et al., 2019; Li, Wu & Wang, 2020; Liu et al., 2020).

Financial time series data encompasses dynamic and nonlinear historical data. This characteristic makes trend analysis task and prediction harder (Cavalcante et al., 2016). While noise and outliers are imperfect observations exist due to some error or abnormal situations taking place during data collection. Noise can be caused by human error or machine error while outliers can be caused by experimental error. These observations originate inconsistency in the data set and may cause poor data modelling along with poor forecasting. It is an important challenge related to preprocessing of data (Yang & Wu, 2006; Grané & Veiga, 2010; Cavalcante et al., 2016).

Text mining is a process to derive meaningful information from raw text. In text mining, text preprocessing is also required in order to remove garbage from text. Then features are extracted from text. In feature extraction, text is parsed to extract features that best reflect the text contents. In the next step, an optimal subset of features is selected that contains all the relevant information. Moreover, features are represented by transforming the selected features into machine readable format (e.g., document vectors) (Hagenau, Liebmann & Neumann, 2013).

The main difficulty in text mining for extracting facts is unstructured form of data (Cho, Wüthrich & Zhang, 1999). Text mining is still an emerging field and the problem of high dimensionality and ignorance of semantics are not tackled strongly in previous literature (Nassirtoussi et al., 2015). Sophisticated techniques are required to extract useful patterns from online unstructured text as it contains massive information (Sumathy & Chidambaram, 2013).

Alternatively, text mining can be performed using sentiment analysis. There are two main approaches to perform sentiment analysis: corpus based and sentiment dictionary or lexicon based (Taboada et al., 2011). So there is a question that which approach is better to adopt in financial domain?

The processed form of textual and numerical features is aligned and given as input into machine learning algorithms in order to learn the market volatility. In the third phase of the generic methodology, machine learning algorithm models the input data and generates predictive signals. Then these predictive signals are used to evaluate prediction accuracy of the proposed approach. Prediction accuracy is estimated by means of machine learning accuracy measures (Cavalcante et al., 2016).

There are complex relations between textual data and historic market data which can be influenced by hidden factors (Ding et al., 2014). In order to capture the influence of textual data over stocks price history an efficient classifier is required.

There are different opportunities proposed in literature to cater the above identified challenges with their limitations and strengths. Before literature review, next section gives a clear understanding of domain under consideration for its readers.

## Key concepts

This section briefly explains the domain of stock prediction using news analysis and defines the key concepts that provide the foundation for the whole discussion. Firstly, it introduces the domain and terms related to stock market prediction. Secondly, it discusses the stock quotes or financial data, derived features and their use. It thoroughly discusses the techniques that clean textual data and extract significant features in it. Then it discusses the prediction algorithms that exploit the combination of numerical and textual data for prediction. Finally, it discloses the most basic evaluation measures to assess the performance of prediction algorithms.

### Stock market prediction related terms

Stock market is a place where corporations publicly trade their stocks to gain funding in order to expand their business. Stocks can be sold or purchased if their companies are listed in the stock market. For instance, in April 2020, there were 542 companies listed in Pakistan’s stock market known as Pakistan Stock Exchange (PSX): http://www.ksestocks.com/AboutPSX.

Stock is a common term, describing the aggregation of shares in a company. While share is a documented ownership issued by the company. This ownership can be any percentage like 10% or 20% ownership depending on the amount of shares an investor has. If a company makes profit, then all shareholders get share of this profit according to their percentage of ownership and their stock price goes up. In the same way if the company is in loss then all shareholders have share in loss as well and their stock price goes down (Setty, Rangaswamy & Subramanya, 2010).

### Time series data

Time series is a set of numerical data points collected at successive points at regular intervals of time. Mathematically, it can be defined as a set of vectors X(t), t = 0, 1, 2, 3…. where t denotes the time interval.

A time series consists of four components: Trend (T), Seasonal (S), Cyclic (C), and Irregular (I). Trend component (T) is an outcome of long term gradual movement in same direction like increase in prices or pollution. Seasonal component (S) shows short term movement of time series data influenced by seasonal factors like sale of heater in cold weather. Cyclic component (C) shows long term rises and falls that are not of fixed period. Irregular or random component (I) is a short term movement that is unpredictable caused by external factors like war, earthquake, flood, etc. All these four components are combined using additive or multiplicative model to form a time series (Idrees, Alam & Agarwal, 2019).

(1) X(t)=T(t)+S(t)+C(t)+I(t)

(2) X(t)=T(t)∗S(t)∗C(t)∗I(t)

Where X(t) is a time series observation in Eqs. (1) and (2).

Techniques for time series prediction can be classified as statistical techniques and machine learning/artificial intelligence (ML/AI) techniques. Example of statistical techniques are Auto Regressive Integrated Moving Average ARIMA, Linear Discriminant Analysis (LDA), Quadratic Discriminant Analysis (QDA), singular value decomposition (SVD), Dynamic Mode Decomposition (DMD), etc. Examples of (ML/AI) techniques are Support Vector Machines (SVM), Neural Networks (NN), Deep Neural Networks (DL), etc.

### Financial data as a time series

Stock prices are treated as time series data. Stock market provides stock quotes or stock price such as Open, Close, Low, High and Volume etc., along with stock symbol and transaction date. These basic quotes give information such as high and low prices of stock in a day or its change in the value.

These financial indicators can be used directly in prediction models as a dependent or independent variable. Such as Close price is used as a dependent variable or label in prediction models (Lin, Yang & Song, 2009; Rustam & Kintandani, 2019). Moreover new features are also derived from the existing one such as gain in (Garcia-Lopez, Batyrshin & Gelbukh, 2018; Mourelatos et al., 2018).

(3) $$Gain\;=(Close\_Price_{t}\;-\;Close\_Price_{t-1})/\;Close\_Price_{t-1\;}$$

In Eq. (3), Close_Price_t-1_ and Close_Price_t_ are close prices for previous and current day.

Trend is another derived attribute that shows upward or downward movement of a stock’s price over time. Trend is derived using the formula:

(4) $$Close\_Price_{diff}\;=\;Close\_Price_{for\;current\;day}-\;\;Close\_Price_{for\;previous\;day}$$

(5) $$Trend\;=\{\begin{array}{c} \;\;\;0\;if\;Close\_Price_{diff}\;\;\leq \;\;0\\ 1\;otherwise \end{array}$$

In Eq. (4), Close_Price_diff_ is a difference between current and previous day close prices. If obtained difference is less than or equal to 0s it means stock is down or no change in stock and Trend is *0*. If difference is greater than 0, it means stock is up and Trend is *1*, stated in Eq. (5) (Ding et al., 2015; Vargas, De Lima & Evsukoff, 2017; Liu et al., 2018).

On the other side technical indicators are the measures that facilitate stock prediction in order to identify the strength and direction of stock trends. In literature, technical indicators have been used with textual features (Li, Wu & Wang, 2020). Basically, they are derived from stock price data. For instance, Moving Average (MA) is a technical indicator that identifies the direction of current price trend. Moving Average Convergence Divergence (MACD), Relative Strength Index (RSI), and Money Flow Index (MFI) are some of the examples of technical indicators used in literature. For description about technical indicators (Gao & Chai, 2018; Alonso-Monsalve et al., 2020) are suggested.

### Textual data

Before feeding textual data to any machine learning model, it should be preprocessed. Data should be cleansed to remove garbage before feature extraction so that it doesn’t produce garbage when fed into machine learning models.

#### Textual data cleansing

Data cleansing is a basic preprocessing step employed to remove unwanted text. There are many approaches involved in data cleansing, and different approaches lead to different results in the model training phase. Moreover, different kind of data (sound, image, speech, etc.) is cleansed by using different approaches. Some basic techniques related to textual data cleansing are discussed below:

##### Removing unwanted characters

Occasionally, data is scraped from a web page like news, and reviews etc. Scraped data may contain html tags, punctuation, and any character which is not a part of the language. These unwanted characters should be filtered out. In case of tweets, there are hash tags, URLs, mentions, and reserved words etc., that are removed to clean the tweets for further processing (Symeonidis, Effrosynidis & Arampatzis, 2018).

##### Tokenization

Tokenization splits text into meaningful chunks and those chunks are called tokens. A token can be a word or a sentence. Tokenization provides the basic unit for further text processing steps. In Uysal & Gunal (2014), Nassirtoussi et al. (2015) and Symeonidis, Effrosynidis & Arampatzis (2018) tokenization is applied to extract a list of separate words from text.

##### Lowercase

Lowercasing is a very common step of text cleansing. It converts whole text into lowercase so that the same words are merged that reduces text dimensionality. In Uysal & Gunal (2014), lowercasing leads to improvement in accuracy.

##### Removing punctuation

There are some scenarios where punctuation adds extra meaning like tweets’ sentiment analysis. For instance, an exclamation mark may increase the intensity of positive or negative remarks. Hence, removing punctuation in that scenario might reduce the accuracy. For other scenarios, removing punctuation is a common preprocessing task where punctuation doesn’t add extra meaning (Schumaker & Chen, 2009; Uysal & Gunal, 2014; Symeonidis, Effrosynidis & Arampatzis, 2018). For instance, text representation into Bag of Words (BoW) considers multiplicity of a word in text such as a sentence or a document. Moreover, the occurrence of words in a text is used as an input feature to train a classifier. So removing punctuation in case of BoW is significant.

##### Removing stop words

Stop words are considered irrelevant and needless to analyze in text before passing to machine learning algorithms. They are high frequency words but don’t contain significant information for examples articles, conjunctions, prepositions etc. This preprocessing step reduces the dimensionality of term space as well. In literature mostly, stop words are removed before further text processing like in Zhai, Hsu & Halgamuge (2007), Guzman & Maalej (2014), Uysal & Gunal (2014) and Symeonidis, Effrosynidis & Arampatzis (2018).

##### Parts-of-Speech (POS) tagging

In POS tagging, each word of a sentence is assigned a label like noun, verb, adjective etc. In text preprocessing, the purpose of POS tagging is to identify and extract specific words that have some worth in different scenarios. Like for sentiment analysis, only nouns, verbs, and adverbs are extracted in Symeonidis, Effrosynidis & Arampatzis (2018) and similarly nouns, verbs, and adjectives are taken as input feature in Guzman & Maalej (2014). Likewise, noun phrases are extracted for textual analysis in Schumaker & Chen (2009). In Ding et al. (2015), events are extracted from news headlines. Each word in a news headline is labeled as noun, verb, adjective, and adverb etc. Then noun, verb, and object are extracted to form an event representation. Then this event representation is used to predict stock movement using deep learning. In Zhai, Hsu & Halgamuge (2007), words are replaced by their generalized concepts using WordNet, where words are grouped semantically. While POS tagging facilitates in disambiguating the word when assigned to WordNet.

##### Word normalization

In English language, words have different forms like wolf or wolves, talk or talks, and write or wrote. Word's different forms actually represent the common base form of a word because these forms are semantically similar. Word normalization merges all forms into a single base or root form of a word. It reduces the feature space by reducing different forms of a word into a single one. Stemming and lemmatization are two ways in NLP to perform word normalization. Stemming converts different derived forms of a word into its root form, also known as stem, by removing the endings of the words like ‘s’, ’ies’, ’ing’ etc. It is a crude way of word normalization performed by just defining some rules of chopping off some characters at the end of the word (Uysal & Gunal, 2014; Singh & Gupta, 2017; Symeonidis, Effrosynidis & Arampatzis, 2018). It is a widely used method and mostly gets good results (Mejova & Srinivasan, 2011). Lemmatization is another method for word normalization. It merges different forms of a word into a root form, also called lemma, by using morphological rules and vocabularies (Singh & Gupta, 2017). Lemmatization is a comparatively more systematic and effective method than stemming (Symeonidis, Effrosynidis & Arampatzis, 2018). In Guzman & Maalej (2014), it is used to reduce features for sentiment analysis.

#### Textual data processing for feature extraction

There are several methods proposed to analyze textual data. The two common methods to mine information are based on objective and subjective information. Objective information extraction techniques deal with facts in the form of shallow features and structural or event representation. While the other promising technique is about subjective information that is sentiment analysis.

##### Shallow features

BoW, noun phrases, and name entities are examples of shallow or simple features. BoW is the most basic shallow feature based technique used for text mining based stock market prediction problems. In this technique, text is broken into words and each word is considered as a feature (Luss & D’Aspremont, 2015; Garcia-Lopez, Batyrshin & Gelbukh, 2018). In its basic form, order and co-occurrence of words are not considered. N-gram is an adjacent sequence of n words. For instance, unigram where *n* = 1 and each word is considered as a feature is same as BoW (Hagenau, Liebmann & Neumann, 2013). But for bigram and trigram, two and three contagious words are considered as an entity. Moreover, two other feature selection techniques namely, named entities and noun-phrases were explored. In noun-phrases, noun POS is identified by using lexicon. While syntactic rules facilitate recognizing noun phrases. In the later one, a pre-defined categorization scheme such as name of person, organization, location etc., is used in order to locate and classify named entities (Schumaker & Chen, 2009; Nassirtoussi et al., 2014). Word embedding is another form of representing text vocabulary. It is a dense representation for text where words that have the same meanings have similar representations (Garcia-Lopez, Batyrshin & Gelbukh, 2018).

##### Event

An event is a specific type of knowledge that can be extracted from text and contains entity-relation information. Formally, an event is defined as E = (A,P,O,T), where P is the action, A is the actor that performs the action, O is the object on which the action was performed and T is the timestamp used for aligning textual data(news) with numerical data(stock price) (Ding et al., 2014). For example, the structure representation of the event “19 February 2014—Facebook buys WhatsApp for $19 billion.” is represented as: “(Actor = Facebook, Action = buys, Object = WhatsApp, Time = 19 February 2014)” (Ding et al., 2015).

##### Event extraction approaches

There are three approaches for event extraction namely data-driven which relies on large corpus of data, knowledge base which makes use of domain knowledge, and hybrid approach where both techniques are combined. All three approaches are listed in Table 1. Data-driven approaches require large text corpus and are based on quantitative approaches to automate text processing, for instance linear algebra, probability theory and information theory. All approaches focus on determining statistical relation without considering semantics explicitly. Since data-driven approaches do not rely on domain knowledge, no expert knowledge is required (Hogenboom et al., 2016).

**Table 1:** Event extraction techniques and their limitations (Adapted from Hogenboom et al. (2016)).

Event extraction techniques	Limitations
Data-driven event extraction	Requires large corpus for training Does not deal with meaning explicitly Does not support result interpretability.
Knowledge-driven event extraction	Requires linguistic and domain knowledge Defining and maintaining patterns express domain knowledge
Hybrid event extraction	Requires linguistic and domain knowledge Results interpretability is difficult. Requires high expertise to tackle complexity raise caused by combination of multiple techniques

Knowledge-driven event extraction methods use domain knowledge in the form of ontologies and patterns that state rules. There are two types of patterns used in knowledge-driven event extraction. The first one is a combination of lexical representation and syntactic information. While the second one is more expressive, and combines lexical representation with syntactic and semantics information. Semantics are usually added by means of ontologies. Due to the use of patterns, knowledge-driven approaches require less training data and results are interpretable and traceable (Hogenboom et al., 2016).

The hybrid approach is a combination of data-driven and knowledge-driven approaches. When domain knowledge is not enough, statistical methods are integrated for compensation. While result interpretability is difficult due to less expressive patterns. So the combination of multiple techniques increase the complexity and require expertise (Hogenboom et al., 2016).

##### Significance of event embedding

Structured representation of events increases sparsity, which most probably decreases the predictive power (Ding et al., 2015). In literature, this issue was solved by using event embedding, which are dense vectors. Event embedding uses the principle that syntactically or semantically similar events should comprise similar vectors. They are used to reduce sparsity due to structured representations of events and capture both the syntactic and the semantic information among events.

##### Sentiment analysis

Sentiment analysis mines the class of emotion from text and assigns a score. The process of sentiment analysis uses NLP and machine learning based approaches. Initially, it breaks down the text into parts like document, paragraph, sentence, phrase, or a word according to required granularity. Then the process of sentiment analysis suggests appropriate sentiment class along with score for the part of text under consideration.

There are two approaches to perform sentiment analysis on textual data. First method uses machine learning algorithm on labeled text in order to create a trained model. Then the train model is used for unseen data to perform sentiment analysis. Second approach is based on rule based sentiment analysis. These rules are also known as sentiment lexicon and inferred by language experts. In this method, text is tokenized into words. Then some preprocessing steps like stop words removal and punctuation removal etc. are performed in order to cleanse the data. Filtered words are classified as positive, negative, or neutral class on the basis of their corresponding intensity measures (Taboada et al., 2011).

Sentiment dictionary or lexicon are constructed using two approaches. In first approach, dictionaries are constructed manually by experts where they also suggest rules to analyze sentiments. For instance, Vader (Hutto & Gilbert, 2014), Harvard IV (HIV4) (http://www.wjh.harvard.edu/~inquirer/homecat.htm), and Loughran and McDonald (LM) (Loughran & McDonald, 2011) are sentiment dictionaries constructed using first approach. Second approach is semi-automated and constructs sentiment dictionary in two steps. In the first step it adopts the same approach but for small dataset where experts manually perform sentiment analysis. Then automated extension methods are applied to construct sentiment dictionary. SentiWordNet 3.0 (Baccianella, Esuli & Sebastiani, 2010) and SenticNet 5 (Cambria et al., 2018) are the examples of sentiment dictionaries constructed using semi-automated approach.

Sentiment dictionaries can be further categorized on the basis of their domain. Like there are general purpose sentiment dictionaries like Vader and HIV4 that can be used in any domain. Domain Specific sentiment dictionary is another type like LM sentiment dictionary which is constructed for financial domain (Li, Wu & Wang, 2020).

Data analytics is a process to examine dataset in order to draw conclusions about the information they contain. There are commercial news analytic vendors like Thomson Reuters and RavenPack who convert the news text into sentiment scores. In Allen, McAleer & Singh (2017) and Deveikyte et al. (2020) news sentiment scores are developed by Thomson Reuters and RavenPack. These sentiment scores are used to study the relationship between financial news sentiment scores and stock prices data.

### Machine learning algorithms

Machine learning enables system to automatically learn and improve itself from experience. The learning procedure extracts patterns from available data and for unseen data these patterns are used for making predictions. The learning procedure is divided into supervised and unsupervised learning. In supervised learning data used for learning is properly labelled or classified. While in unsupervised learning data is not labelled and it is explored to identify the hidden patterns (Tabares-Soto et al., 2020). In this section some supervised learning techniques are discussed which are commonly used in literature for stock prediction using textual analysis.

#### Naive bayes (NB)

Naïve bayes is a probabilistic machine learning algorithm. It is based on Bayes theorem. It is a simple but powerful prediction algorithm used mostly in text classification tasks such as spam filtering. Classification is based on the joint probabilities of features and classes. It is called naïve because features passed into the model are independent to each other. That is changing the value of one feature, does not directly influence or change the value of any of the other features used in the algorithm (Dada et al., 2019). In text mining where the number of features are large, naïve assumption simplifies learning and NB outperforms SVM and KNN (Yang & Liu, 1999).

#### K-nearest neighbors (KNN)

It is one of the simplest supervised machine learning algorithms with no or very minimal training phase. It does not make any underlying assumptions about the distribution of data because it is non parametric. Classifications are made using training data by matching the test example with each and every training example. That is, a complete training dataset is used to predict class for every test example. This makes KNN inefficient in terms of time and memory. Class is assigned to test instance on the bases of K similar nearest training instances where the optimum value of K is mined through tuning (Groth & Muntermann, 2011; Dada et al., 2019).

#### Support vector machines (SVM)

Support Vector Machines are supervised machine learning algorithms. The main notion behind SVM is about finding a hyperplane that best classifies the data points. There might be many candidate hyperplanes for data segregation but the best one has a maximum margin that is the maximum distance between data points and a hyperplane. Maximizing marginal distance enhances classification accuracy when test data points are classified. Another, promising feature of SVM is that they shift data points into higher dimensional space if they are not separable in present dimensional space. SVM are slow but provide higher accuracy than other prediction algorithms and this feature makes SVM a preferable choice for text categorization related applications (Cho, Wüthrich & Zhang, 1999; Dadgar, Araghi & Farahani, 2016; Dada et al., 2019).

#### Artificial neural network (ANN)

Artificial Neural Network is a complicated but powerful machine learning technique that imitates the human brain functionality. ANN is a group of neurons, the basic processing unit, which are interconnected and communicate with one another through weighted connections. Neurons take inputs, multiply these inputs with their corresponding weight and add them to get a value, and finally pass this value to an activation function to produce an output. These neurons are arranged in a manner so that they form a layer. ANN consists of three layers namely input, hidden, and output. Each layer consists of at least one neuron. There might be more than one hidden layer. Neural networks are trained using large number of iterations. One iteration to train a neural network has two steps. In the first step, neural network receives input through the input layer and passes it to the hidden layer(s), then the output of the hidden layer(s) moves towards the output layer. Finally, output is generated by the output layer of the neural network. This is called feed-forward propagation. The estimated output is compared with actual output in order to calculate the prediction error. Second step is called backpropagation step. It starts by adopting gradient based method. This gradient based method is used to find optimized weight values in neural network. Gradient based method calculates derivative of error with respect to each weight using chain rule. Backpropagation step is completed when all weights are updated. ANN is a powerful machine learning algorithm used as a text classifier in literature (Groth & Muntermann, 2011; Dada et al., 2019). In Fig. S1, structure of ANN is illustrated.

#### Deep learning

Deep learning is a sub field of machine learning, based on ANN. It is a new emerging area, also known as Deep Neural Network (DNN). The promising feature of deep learning is that it can learn features from data directly using multiple non-linear hidden layers. Deep learning models are able to solve sophisticated problems and their effectiveness becomes more prominent as training data increases. These models exploit the computation power of modern Central Processing Unit (CPU) and Graphics Processing Unit (GPU) to facilitate heavy processing.

Deep learning models face a challenge of vanishing gradient which slows down model’s training and in worst case stops it. This problem arises in deep neural networks due to deep hidden layers and it makes difficult to learn weights of earlier layer. According to chain rule, derivatives are multiplied to each other from last layer to first layer in order to compute the derivative of initial layers. If derivatives are small values, multiplication makes it so small. In this situation weights are not updated effectively which increases inaccuracy in a model prediction (Charniak, 2019; Hu, Zhao & Khushi, 2021).

##### Convolutional neural networks (CNNs)

CNN is a deep learning model inspired by human vision mechanism and used across different applications especially in image processing and video processing tasks. Later, CNNs are enhanced for text classification. For instance, in Dada et al. (2019), CNN is successfully used for email spam filtering. The basic architecture of CNN is composed of several layers, for instance input layer, convolution layer, pooling layer, and fully connected layer. It is illustrated in Fig. S2.

Input is fed through input layer in the form of three dimensional array. Convolution layers are used to extract features. While former convolution layer extract features more abstractly than later convolution layer. These layers have filters that are small matrices to perform convolution operation. Pooling layer reduces the spatial dimension of input that is why it is used after convolutional layers. Convolution and pooling layers reduce the input dimension which in turns speed up computation. Last layer is fully connected where each neuron is connected to the neurons in the previous layer. An important feature of CNN is weight sharing which makes it less complex than fully connected neural network (Goodfellow et al., 2016; Charniak, 2019; Hu, Zhao & Khushi, 2021).

##### Recurrent neural network (RNN)

RNN architecture is used to learn from sequential data. ANN models are used for traditional predictive analysis is not suitable for sequential data because observations in sequential data is not independent to each other. Traditional neural network treats each input as an independent entity. RNN preserves previously processed observations using hidden state and uses it along next observation going to be processed. Information in RNN travels through loop which makes it possible to use same parameters and reduces the parameter complexity than other NN models. But RNN doesn’t support long-term memory and faces vanishing gradient problem (Goodfellow et al., 2016; Charniak, 2019; Hu, Zhao & Khushi, 2021). The architecture of RNN is shown in Fig. S3.

##### Long short term memory (LSTM)

LSTM is a variant of RNN. It is also designed to support sequential data processing. It tackles the shortcoming of RNN like vanishing gradient and no support for long-term memory by introducing its gating mechanism. It has three gates which are input gate, output gate, and forget gate. Three types of information are passed into LSTM cell, the current input, hidden state (short-term memory), and cell state (long-term memory). The forget gate decides which information in cell state should be kept or discarded. While the input gate is responsible for what new information should be stored in cell state. The output gate receives current input, previous hidden state, and newly computed cell state in order to generate new hidden state and output for current input observation in sequence (Goodfellow et al., 2016; Charniak, 2019; Hu, Zhao & Khushi, 2021). Architecture of LSTM is depicted in Fig. S4.

### Evaluation measures

The performance of the classification algorithms is measured using accuracy, precision, recall, and F1-score (Li, Wu & Wang, 2020). In order to understand these basic metrics, there are some key terms which are the primary building blocks of these metrics. For instance, a true positive (TP) is a number of correctly predicted positive classes by the prediction model. Similarly, a true negative (TN) is the number of correct predictions for a negative class. A false positive (FP) is known as the number of incorrect predictions of a positive class made by the prediction model. While a false negative (FN) is an outcome where the model creates incorrect prediction for the negative class (Patel et al., 2015). By using these key terms, evaluation metrics can be defined as:

#### 

##### Accuracy

Accuracy is the measure of correctly predicted instances divided by total number of instances.

(6) $$Accuracy=\frac{\left(TP+TN\right)}{\left(TP+TN+FP+FN\right)}$$

##### Precision

Precision is the ratio between correct predictions for a class and total number of predictions for a class.

(7) $$Precision_{\left(Positive\;Class\right)}=\frac{TP}{\left(TP+FP\right)}$$

(8) $$Precision_{\left(Negative\;Class\right)}=\frac{TN}{\left(TN+FN\right)}$$

##### Recall

Recall is the ratio between correct prediction of a class and total number of actual predictions of a class.

(9) $$Recall_{\left(Positive\;Class\right)}=\frac{TP}{\left(TP+FN\right)}$$

(10) $$Recall_{\left(Negative\;Class\right)}=\frac{TN}{\left(TN+FP\right)}$$

##### F1-score

F1-Score is also known as F-measure. It performs well if the prediction algorithm is dealing with uneven class distribution. It is a weighted average of Precision and Recall measures.

(11) $$F1-Score=2\;*\;\;\frac{\left(Precision*Recall\right)}{\left(Precision+Recall\right)}$$

The above measures formulated from Eq. (6) to Eq. (11), are particularly used to evaluate performance of classification algorithms. On the other hand, there are other basic measures which are used to measure the performance of regression algorithms where predicted value is a continues value. Evaluation metrics for regression problems identify the difference between actual and predicted value (Gao, Chai & Liu, 2017; Jin, Yang & Liu, 2019). Some of the basic evaluation measures for regression problem are discussed below:

##### Mean square error (MSE)

Mean square error is a mean squared difference between actual and predicted values identified by regression task.

(12) $$MSE=\frac{\sum_{i=1}^{\;\;n}\left(Predicted_{i}-Actual_{i}\right){\;}^{2}}{n}$$

##### Root mean square error (RMSE)

Root mean square error is a square root of mean squared difference between actual and predicted values.

(13) $$RMSE=\sqrt{\frac{\;\;\sum_{\;i=1}^{\;\;n}\left(Predicted_{i}-Actual_{i}\right){\;}^{2}}{n}}$$

##### Mean absolute error (MAE)

Mean absolute error is a mean of absolute difference between actual and predicted values.

(14) $$MAE=\frac{\sum_{i=1}^{\;\;n}\left|Predicted_{i}-Actual_{i}\right|}{n}$$

Where

***n***= number of observations

For further detail about evaluation measures for regression task (Hyndman & Koehler, 2006) is suggested.

## Literature review

This section briefly outlines the research on stock prediction techniques. It summarizes the techniques that only consider numerical financial data for stock prediction. Then it discusses the feature extraction from textual data. It also summarizes the prediction algorithms that exploit the combination of numerical and textual data for prediction.

### Forecasting stock trend using time series data

Time series analysis for stock prediction using statistical approaches and machine learning-based approaches have been adopted for many years. Both approaches have their own limitations and strengths. Initially, researchers adopted statistical approaches like authors in (Huang, Nakamori & Wang, 2005) identified the financial movement direction of the NIKKEI 225 index by using different techniques. Forecasting performance of Support Vector Machine (SVM) is better than Linear Discriminant Analysis (LDA), Quadratic Discriminant Analysis (QDA) and Elman Backpropagation Neural Networks (EBNN). Combination of all these forecasting models further improves forecasting accuracy. A major limitation of this work is that the authors have used weekly price movement. While daily price movement would have given more insight in perceiving price volatility.

Ou & Wang (2009) examined the Hang Seng index of Hong Kong stock market. Experiments are performed using ten data mining techniques to predict price movement of the Hang Seng index of Hong Kong stock market. These techniques include k-Nearest Neighbor (k-NN) classification, LDA, QDA, Naive Bayes (NB), Neural Network (NN), Logit model, Tree based classification, Bayesian classification with Gaussian process, SVM and Least Squares SVM (LS-SVM). It is observed that each of the algorithms has its own limitations. The prediction model accuracy depends on input representation, feature selection, and prediction algorithm. SVM and LS-SVM performed best among the other models. Their empirical data is detailed and gives the right direction to further enhance the strength of prediction by combining these techniques. This paper provides a useful baseline for further investigation to improve prediction performance.

Dynamic Mode Decomposition (DMD) is relatively a newer approach to analyze time series data. DMD takes advantage of coherent structures in time series. These coherent structures are called DMD modes and give more physical insight than curve fitting. The application of trading strategy based on DMD is proposed in literature by many researchers to identify evolutionary patterns from stock price data (Mann & Kutz, 2016). The strength of DMD is demonstrated in Cui & Long (2016) for Chinese A-share stock market for modeling the market patterns well in sideways market. Hua et al. (2016) extract cyclic activity and price prediction from daily stock price data using DMD method. In Kuttichira et al. (2017) adopted DMD for short term stock price prediction. They used minute-wise data of National stock market for predicting the price in next few minutes. Prediction was performed for intra and inter sector companies and evaluated using mean absolute percentage error. Time series analysis using DMD based approach is useful because DMD is an effective and computationally efficient approach but it is lacking in improving prediction performance for unexpected changes like stock split.

Sun & Li (2012) proposed a new SVM ensemble method for Financial Distress Prediction (FDP). Their main contribution is the technique for the selection of SVM ensemble’s base classifiers on the basis of individual SVM classifier’s performance. It was proved by experimental outcomes that the SVM ensemble was significantly better than individual SVM classifier. They have relied on financial ratios and ignored the importance of market factors such as firm size, and volatility etc. in predicting financial distress. Moreover, they have used a small dataset of five years for this study. Huang, Huang & Han (2012) adapted Support Vector Regression (SVR) as a forecasting technique for financial time series data. Furthermore, SVR kernels are built using wavelet functions. Comparative analysis showed that it is better than standard kernel function. Their proposed technique improved prediction performance but a use of wavelet function as a de-noising technique along with SVM is missing. Comparative analysis between both uses of wavelet function will give more insight for better utilization in dealing with time series data.

Cheng & Wei (2014) proposed SVR with Empirical Mode Decomposition (EMD) to predict stock price. Then in order to show its performance, comparison with a simple SVR model and Autoregressive (AR) model is performed. EMD is an adaptive approach that can decompose nonlinear time series into intrinsic mode functions (IMF). It can suppress continuous noise. In Yujun, Yimei & Jianhua (2020), EMD and its variant Ensemble EMD, which are better in dealing noise, are adopted to decompose a time series into subsequences. Then LSTM is used to learn and predict from these subsequences. Finally, subsequences prediction results were combined to obtain the estimation of original time series. This is a good attempt and they have taken the advantage of statistical and machine learning methods as a hybrid approach. Moreover, other preprocessing techniques can also be employed in comparison with EMD to better understand its strength. Furthermore, the use of technical indicators can enhance the prediction performance.

The financial time series data has characteristics of nonlinearities and discontinuities which add complexity in forecasting techniques. Artificial Neural Network (ANN) is getting popular because it can handle the complex characteristics of financial time series data. The Istanbul Stock Exchange stock prices volatility is forecasted using ANN and SVM prediction models. Comparisons show that ANN achieves better results as compared to SVM (Kara, Boyacioglu & Baykan, 2011). However, the use of fundamental factors as input variables besides technical indicators are missing. (Ticknor, 2013) used Bayesian Neural Network (BNN), a kind of ANN with tangent sigmoid function as a hidden layer transfer function to predict stock price. In this study, very limited number of technical indicators are used as a set of input features. While there is still a space for further investigation to analyze prediction performance on different combinations of technical indicators.

Liu & Wang (2012) concentrated on the prediction of price fluctuation in the stock market and proposed a three layered improved Legendre Neural Network (LNN) model. Further improvement can be made in random time strength function for different financial markets to increase the prediction accuracy. Shahpazov, Velev & Doukovska (2013) examined three prediction models to predict Bulgarian stock market. They used Multi-Layer Perceptron (MLP), Radial Basis Function (RBF) neural network, and General Regression Neural Network (GRNN). While the performance of GRNN was better than others. When dealing with neural networks, dataset size plays an important role in optimizing model’s performance. But the dataset incorporated by them is limited in size. Wang & Wang (2015) proposed Stochastic Time Effective Function Neural Network (STNN) for stock prediction. Principal Component Analysis (PCA) is used to identify principal components. Furthermore, in order to ensure PCA-STNN predictive performance, the model is compared with Back Propagation Neural Network (BPNN), PCA-BPNN and STNN. But the predictive performance is not satisfactory when there is a large fluctuation period in time series. Nelson, Pereira & De Oliveira (2017) adopted deep neural networks for the stock market prediction. Their work is based on LSTM which can be further enhanced by adopting different preprocessing techniques. Arratia & Sepúlveda (2019) adopted the CNN model. The financial time series data is converted into images and then passed into CNN which produced improved classification accuracy for stock market prediction. The work performed by them is encouraging. However, they have used cross validation for time series data according to the work of (Bergmeir, Hyndman & Koo, 2018) although the time series under consideration was not artificial and stationary.

The above literature review is summarized in Table S1. This review exposes the superiority of SVM and ANN over other machine learning techniques. It is deduced that statistical methods don't perform better time series analysis as compared to ML approaches like SVM and ANN. Furthermore, DL based techniques like CNN and LSTM tackle nonlinearities and volatilities better than shallow learning based ML techniques.

### Stock trend prediction using textual data

Unstructured form of textual data makes it difficult for data mining techniques to mine information from text. Moreover, these text mining techniques can be further classified as fact mining and opinion mining techniques. In this section, literature regarding text processing techniques is discussed and divided into three groups: shallow feature based text processing, event extraction based text processing, and sentiment analysis.

#### Shallow feature based text processing

Cho, Wüthrich & Zhang (1999) proposed text processing techniques based on keyword record counting. They used a fixed set of news stories. In these news articles, they searched around 400 keywords which were provided by market experts. They only considered words statistics and ignored their semantics. They examined several text processing methods and their effectiveness in forecasting financial markets.

Schumaker & Chen (2009) analyzed financial news articles using shallow features based textual representation approaches like Bag-of-Words (BoW), noun phrases, named entities, and proper noun schemes. They input different feature types in SVM classification to study their impact and recognize the superiority of proper noun schemes as compared to other text processing techniques. But they used small dataset that is not enough for in depth analysis.

Dadgar, Araghi & Farahani (2016) proposed a news classification approach using Term Frequency–Inverse Document Frequency (TF-IDF) for feature extraction and SVM as classification scheme. They compared it with other classification methods and found desirable results using their proposed approach. However, text preprocessing phase can be improved further by using word normalization techniques.

Li et al. (2016) proposed a stock market prediction scheme using Extreme Learning Machine (ELM) for rapid forecasting. ELM is a type of feed forward neural network with one hidden layer. ELM doesn’t employ gradient based methods for parameter optimization. They have used a news filtering scheme but a situation where multiple news for a stock occur in the same time window are not handled properly and discarded from filtered news dataset.

Groth & Muntermann (2011) used BoW and applied NB, k-NN, SVM and NN to learn patterns from text. In order to generalize prediction model, size of the dataset was not enough. Hagenau, Liebmann & Neumann (2013) used SVM prediction model and input textual feature set along with technical data. They showed that prediction performance is enhanced by using their feature extraction techniques where extracted BoW feature set is refined using market feedback. However, text preprocessing steps are not extensive only stop word removal technique is employed in the text preprocessing step.

Zhai, Hsu & Halgamuge (2007) used two news dataset where one is for general market and the other is for specific stock. Textual features are extracted using BoW and then extracted words are replaced with their high level concept using WordNet. These concepts are weighted using TF-IDF scheme. Selected textual features are combined with technical indicators and passed into SVM model for predictions but experiments are performed on small dataset.

Luss & D’Aspremont (2015) utilized textual and numerical data for intraday price prediction. They employed Multiple Kernel Learning (MKL) for price prediction and BoW to extract text features. These features are selected using dictionary of word’s stem form that reduced dimensionality of feature vector. But dictionary contained small set of words that limited the prediction performance.

Vargas, De Lima & Evsukoff (2017) combined CNN with LSTM and used word and sentence embedding for stock prediction. They have compared their results with (Ding et al., 2015) and showed that performance became slightly better for word and sentence embedding. They have used hybrid prediction model and technical indicators but preprocessing step for financial time series is missing. Furthermore, the comparative analysis of experimental results using CNN and CNN combined with LSTM, showed that features with enhance semantic significantly contribute in improving prediction accuracy.

In Garcia-Lopez, Batyrshin & Gelbukh (2018), the relationship between tweets and stock trend is captured while BoW and word embedding are used as a textual representation. BoW vocabulary size is justified by analyzing results for different vocabulary size but Word2Vec embedding size parameter is not tested for different values. It is shown that word embedding outperformed BoW. Yun, Sim & Seok (2019) used titles of news articles in Korean language and extracted features using word embedding. Features were passed into the CNN model and it produced 53% accuracy. In this research, authors have not discussed about hyper parameter’s tuning. The discussed literature about shallow features is summarized in Table S2.

#### Event extraction based text processing

Event extraction approaches are based on large data corpus and do not depend on domain expertise. These approaches are used in many domains like finance, security, and war etc. In data-driven event extraction, clustering is performed to group documents or sentences that refer to the same event (Naughton, Kushmerick & Carthy, 2006; Tanev, Piskorski & Atkinson, 2008). Ding et al. (2014) and Ding et al. (2015) used data-driven event extraction based stock prediction techniques. Structured events are extracted using OpenIE technology. Extracted events are generalized using two ontologies (WordNet and VerbNet). Then linear prediction models are compared with non-linear prediction models for capturing the hidden relationship between financial events and stock trend. They found that events based feature set performed better than BoW based feature set using non-linear prediction models. But the sparseness of event based features set limited the prediction accuracy. In their next work, extracted events are further processed by using event embedding. These embedded events are used to produce textual features for CNN prediction technique. It is observed that event-embedding based document representation improves the prediction accuracy more than discrete event based document representation. The proposed event embedding technique is based on word embedding of the elements of an event. This event embedding technique cannot capture the relationship between two semantically same events if word vectors are not same. In Ding et al. (2016), the issue is addressed by introducing background knowledge. But they should extend their work for financial domain related knowledge graph. Deng et al. (2019) employed the similar technique for event embedding using knowledge graph to refine event embedding. For prediction, Temporal Convolutional Network (TCN) is employed that outperformed other deep learning models especially for abrupt changes of stock trend. However, accuracy is not the only metric to show the worth of prediction model. The prediction model should also be analyzed for the time and memory it takes for training.

Event extraction based on expert knowledge with small data corpus improves search performance of information extraction technique (Borsje, Hogenboom & Frasincar, 2010; Hogenboom et al., 2013). Chen et al. (2019) extracted fine grained events automatically using a finance event dictionary built by domain experts. They proposed a professional financial event dictionary that contains all main financial events along their roles, and trigger words. By using this dictionary, events are extracted along their semantics automatically. This fine grained event significantly improves the prediction performance. However, the shared effect of stock and news is distributed between parameters of prediction model. So, it is difficult to extract this learned effect in order to use it in other financial problems.

For pattern based event extraction, lexico-semantic patterns are better than lexico syntactic (IJntema et al., 2012). Author proposed lexico-semantic pattern language that make use of patterns to identify semantics from text. It is preprocessed before pattern matching in text. They performed experiments for finance and the political domain and found results with good precision and recall. But the extracted events are not timestamped, which is a mandatory feature in other domains like financial domain especially for trend prediction.

In Nuij et al. (2013) a framework is proposed for automatic extraction of news events from news messages. Knowledge based event extraction is performed using ViewerPro tool. Then extracted events impact and technical indicators are used in stock trading strategies. These strategies take the form of rules that use technical indicators with news variables. Genetic programming is used to reveal intricate rules based on news-based signals and technical indicators. Extracted events are not analyzed thoroughly like understanding the relationship between multiple events occurring in same time window so that their collective impact can be inferred on stock trend.

Many knowledge based event extraction techniques require data-driven processing steps like initial clustering hence these approaches are combined with their pros and cons. Hybrid event extraction techniques are used in many domains like biomedical, politics, weather etc., (Jungermann & Morik, 2008; Björne et al., 2010). However, hybrid approach increases complexity by utilizing multiple techniques and requires high expertise to deal with. Event extraction based text processing is summarized in Table S3.

#### Sentiment analysis based text processing

Stock prediction using sentiment analysis is an attractive area of research as it gives deeper analysis of textual data. In Sehgal & Song (2007), Yahoo financial message board is used as a source of textual data for predicting stock trend. They inferred public sentiments from web messages and proved its correlation with stock trend. Naïve bayes, decision tree, and bagging algorithms are used as prediction algorithm. They also added an important contribution in terms of *trust value* parameter. It is calculated using author’s past performance related to correct predictions. On the basis of trust value unreliable sentiments are filtered which further enhances prediction accuracy. However, they have only considered past day web sentiments while there are web sentiments that have long term correlation with stock value.

It is shown by experiments in Wu et al. (2012) that the use of sentiment analysis based features along with technical indicators enhances the prediction performance. They used pointwise mutual information (PMI) measurement to extract sentiment analysis based features. PMI measures strength of semantic association between words and seed words from positive and negative class. The proposed technique for features extraction from stock news captures effective features which enhances prediction performance. But technical analysis can be improved further by examining different combinations of technical indicators.

De Fortuny et al. (2014) proposed a stock prediction model with SVM as a classifier. The input set is comprised of technical indicators and textual features. Textual features are extracted using BoW and sentiment analysis for different scopes of textual data. However, sentiment analysis only considered adjectives in text. By performing multiple experiments, they have given strong prove that stock prediction model performed better than random guessing.

Initially, sentiment analysis is tackled as a standard document classification problem, but soon it was realized that it requires some established knowledge base in the form of rules and vocabulary. Besides machine learning based sentiment analysis, lexicon based sentiment analysis is considered as a key resource for sentiment analysis.

In the beginning, sentiment lexicon was created manually with small data size (Loughran & McDonald, 2011; Hutto & Gilbert, 2014). There is a need to automate the creation process of sentiment lexicon in order to increase lexicon data size. SenticNet5 (Cambria et al., 2018) contains around 100k concepts along with relationships between these concepts. They automatically discovered conceptual primitives from text and linked them to commonsense concepts and named entities using symbolic and sub-symbolic AI. Recent studies show that these large scale lexicons provide significant improvement in performance. In Jovanoski, Pachovski & Nakov (2016), using mid-sized manually crafted lexicon as seeds, a large scale lexicon is built using bootstrapping. It is shown that mid-sized seeds lexicon produces high quality lexicon than using small-sized seed lexicon. By using Sentprop, a framework to induce sentiment polarity for specific domain, (Kreutz & Daelemans, 2018) adapted a general purpose sentiment lexicon for use in one specific domain.

Sentiment lexicon is a powerful tool that provides sentiment analysis an establish foundation. In Deng et al. (2011), SentiWordNet 3.0 was used as a sentiment lexicon to perform sentiment analysis for news and comments scrapped from social network. They performed technical analysis on stock price data for feature extraction. Finally, all types of features are input into multiple kernel learning regression model (MKL) to perform prediction. Evaluation results showed that multiple type of features enhanced prediction performance. For sequence learning, neural network based model like LSTM performs better than MKL.

Li et al. (2014) have adopted Harvard IV-4 dictionary and Loughran-McDonald financial dictionary to construct the sentiment dimensions. News articles are then quantitatively projected onto sentiment dimensions. Prediction models with sentiment analysis outperformed the BoW models at index, sector, and stock level. Performance difference between Harvard IV-4 dictionary and Loughran-McDonald financial dictionary is not noticeable. Techniques should be employed to automatically expand sentiment dictionaries. In Li, Wu & Wang (2020), technical indicators are combined with news sentiment scores. Sentiment analysis is performed using general purpose and domain specific sentiment lexicon. Prediction model showed better result with domain specific lexicon than other lexicons. Multiple news sentiments within the same day are averaged to input in prediction model. But there should be a way to identify strength of each news so that their weighted average can be taken.

Allen, McAleer & Singh (2017) used DJIA news sentiment scores provided by Thomson Reuters. They analyzed the relationship between DJIA component companies stock prices and financial news sentiment scores using entropy based measures. In Deveikyte et al. (2020), news headlines from FTSE100 is obtained from RavenPack. Furthermore, tweets and news stories for FTSE100 are also collected from other sources. They have assessed the relationship between stock market movement and sentiment score. They have also conducted a correlation analysis using Granger’s causality test. Both articles revealed the significance of sentiment score in estimating stock market behavior. However, dataset size was not enough for reliable results.

### Machine learning techniques for stock prediction using numerical and textual data

In literature, machine learning based stock prediction techniques are divided into shallow learning and deep learning techniques. Research papers for stock prediction are discussed in earlier section in the context of text mining approaches. In this section, these research papers are outlined under machine learning categories.

#### Shallow learning techniques for stock prediction

Shallow Learning techniques provide predictive models with very few numbers of composition layers. It requires features that have already been processed well. It can work well with small dataset (Pasupa & Sunhem, 2016). SVM and Artificial Neural Network (ANN) with one or two incorporated hidden layers are examples of shallow learning techniques. In Schumaker & Chen (2009) and Dadgar, Araghi & Farahani (2016), news classification approaches are proposed using different shallow features based textual representation and SVM machine learning models. Groth & Muntermann (2011) used NB, k-NN, SVM and NN for text classification. Zhai, Hsu & Halgamuge (2007), Hagenau, Liebmann & Neumann, 2013 and Luss & D’Aspremont (2015) used SVM model for stock prediction. Garcia-Lopez, Batyrshin & Gelbukh (2018) used Naive Bayes, Support Vector Machines, K-nearest neighbors, Logistic Regression, and multilayer perceptron. BoW and word embedding are used for text representation while classifiers performed better with word embedding than BoW.

#### Deep learning techniques for stock prediction

Deep learning techniques as compared to ANN, have several hidden layers. Deep learning algorithms require a big dataset in order to tackle over-fitting as well as a high performance computing unit like GPUs (Pasupa & Sunhem, 2016). The promising feature of deep learning techniques is that it can extract features from data through learning. CNN and Recurrent Neural Network (RNN) are types of deep learning techniques. Vargas, De Lima & Evsukoff (2017) proposed a Recurrent Convolutional Neural Network model (RCNN) to take advantage of both models. CNN better extracts semantic information from text and RNN is better in catching context information. The results showed that the proposed model RCNN performance is better than CNN. In Ding et al. (2015), evaluation results showed that CNN is superior to standard feedforward neural networks and SVM. Deng et al. (2019) proposed Knowledge Driven Temporal Convolutional Network (KDTCN) model based on 1-D Fully-Convolutional Network (FCN) architecture. KDTCN outperformed other advanced models for stock prediction. In Jin, Yang & Liu (2019), a hybrid model is proposed that used stock prices data and investors sentiments. The proposed model adopted EMD as a frequency decomposition technique in order to deal with non-stationary stock price time series data. While LSTM with attention mechanism verified the superiority of the hybrid model in terms of prediction accuracy.

## Discussion

Stock market prediction is automated using two approaches: technical analysis and fundamental analysis. Technical analysis relies on historic price data, volume of transactions and their derived attributes. While fundamental analysis not only considers technical data but also incorporates economic and political factors that have direct impact on stock market. These factors can be captured from textual data in the form of news, tweets, company performance reports, etc.

The discussion starts in the context of technical analysis by considering stock price data as a time series data that contains non-linearity and volatility or noise. Initially, technical analysis adopts statistical approaches for stock prediction. These statistical approaches are limited in perceiving non-linearity, noise and dynamics between stocks. Instead machine learning and AI based techniques especially deep learning has brought substantial advancement to deal with stock price data. However, despite this feature of machine learning techniques, statistical methods are not entirely discarded from the set of opportunities. Likewise, EMD and its variants are used in literature as a preprocessing of non-linear and noisy data (Yujun, Yimei & Jianhua, 2020).

Many de-noising techniques have been proposed to filter time series data like Independent Component Analysis (ICA) and Wavelet Transform (WT) (Wang et al., 2011; Dai, Wu & Lu, 2012; Liang et al., 2019). Furthermore, segmentation is a pre-processing step that represents time series with less data and identifies technical patterns which facilitates data analysis (Cavalcante et al., 2016). This discussion about analyzing stock price data deduces that stock price data should be analyzed by combining the strength of statistical and deep learning techniques. Forecasting using hybrid approaches improves accuracy significantly.

On the other hand, fundamental analysis deals especially with textual data. There are two types of approaches that mine information from textual data. First category is related to fact finding techniques and the other one is opinion mining techniques. In literature review, two prominent approaches are discussed for extracting facts from text that are shallow and event based features. Most earlier research work adopted text processing using shallow features like bag-of-words, noun phrases, and named entities etc. These techniques are basically different representations of text vocabulary with high dimensionality and without semantics (Schumaker & Chen, 2009; Groth & Muntermann, 2011). Then some techniques are employed for shallow feature’s dimensionality reduction for instance, knowledge bases and generalized concepts (Zhai, Hsu & Halgamuge, 2007; Luss & D’Aspremont, 2015). Word embedding which is another text representation technique reduces the dimensionality by allowing words with similar meaning to have similar representation (Vargas, De Lima & Evsukoff, 2017; Garcia-Lopez, Batyrshin & Gelbukh, 2018). Later on, an entity relationship based technique is adopted for information extraction from text. So textual data is represented as structured events which contain entity-relation information (Ding et al., 2014). The use of domain knowledge along with event extraction method refines this process and provides opportunity to use less training data with interpretable and traceable results (Hogenboom et al., 2016; Chen et al., 2019). Moreover, event embedding gives the distributed representation of structured events which significantly reduces the issue of high dimensionality. While the use of knowledge base along with event embedding further refines textual feature extraction process (Ding et al., 2016; Deng et al., 2019).

Alternatively, sentiment analysis is another way to mine information about opinion in text. Using machine learning algorithms, sentiment analysis is performed as a document classification problem (De Fortuny et al., 2014). Another approach adopts sentiment lexicon to identify sentiment score. Sentiment lexicon provides an established foundation and it is mostly adopted for sentiment analysis. While sentiment lexicons for financial domain can enhance stock prediction accuracy like LM (Loughran & McDonald, 2011). But its limited size doesn’t impact well on prediction accuracy. So, there are approaches that automate the process of increasing the size of sentiment lexicon by taking into account existing manually created data as a seed word set (Jovanoski, Pachovski & Nakov, 2016; Kreutz & Daelemans, 2018). In financial domain, lexicon based sentiment analysis is performed to extract features from textual data (Li, Wu & Wang, 2020).

Features extracted from numerical and textual data are aligned and input into prediction algorithm. Deep learning has more potential than shallow learning techniques to capture the complex hidden relationship between textual and numerical data. The promising feature of deep learning techniques is that they can extract features from data through learning. The work of Ding et al. (2015), Ding et al. (2016), Vargas, De Lima & Evsukoff (2017), Chen et al. (2019), Deng et al. (2019) and Li, Wu & Wang (2020) shows the strength of deep learning for prediction along with event base textual representation and sentiment analysis based features (Picasso et al., 2019; Li, Wu & Wang, 2020). Solutions of the challenges in implementing news sensitive stock prediction are discussed in this section and are summarized in Table 2.

**Table 2:** Key challenges and proposed solutions.

Challenges	Descriptions	Solutions
Process financial time series data	Dynamic and nonlinear data with noise and outliers	Data Filtration, segmentation (Wang et al., 2011; Dai, Wu & Lu, 2012; Cavalcante et al., 2016; Liang et al., 2019), Data decomposition (Jin, Yang & Liu, 2019; Yujun, Yimei & Jianhua, 2020)
Process textual data	Unstructured data, Lack of semantics, emotion extraction	Shallow features with dimensionality reduction (Vargas, De Lima & Evsukoff, 2017), Structured representation using event driven approach with embedding and use of knowledge bases (Ding et al., 2016; Chen et al., 2019; Deng et al., 2019). Sentiment analysis using lexicon based approach (Li, Wu & Wang, 2020)
Prediction technique	Ultra High dimensional classification problem, identify influence of textual data on stock history	Deep neural network (Ding et al., 2015; Pasupa & Sunhem, 2016; Vargas, De Lima & Evsukoff, 2017; Mourelatos et al., 2018; Deng et al., 2019)

Finally, Table 3 outlines state of the art techniques in the context of news sensitive stock prediction model. By observing this table, it can be deduced that hybrid approaches for feature extraction and prediction perform better by combining strengths of different approaches.

**Table 3:** News sensitive stock market prediction models using state of the art techniques.

Reference	Data Source	Numerical Data	Textual Data	Prediction Techniques	Evaluation metrics
Ding et al. (2016)	S&P 500 through Yahoo Finance, News articles from Reuter’s website from October 2006, to November 2013	Stock price data	Knowledge driven event embedding (KGEB)	KGEB-CNN	Accuracy = 66.93%
Vargas, De Lima & Evsukoff (2017)	S&P 500 index series are obtained through Yahoo Finance, News articles from Reuter’s website from 20-10-2006, to 2-11-2013	Technical data	Word embedding and sentence embedding	Combines LSTM with CNN	Accuracy for word embedding and technical indicator = 61% Accuracy for sentence embedding and technical indicator = 62%
Chen et al. (2019)	Tokyo Stock Price Index (TOPIX), Financial news from Reuters	Stock price data	Proposed financial event dictionary, fine grained event using dictionary	Structured Stock Prediction Model(SSPM), Multi-Task Structured Stock Prediction Model(MSSPM) Using BiLSTM, self-attention and Conditional Random Fields (CRF) etc.	SSPM Accuracy = 66.4% MSSPM Accuracy = 65.7
Deng et al. (2019)	DJIA index from 08/08/2008 to 01/01/2016. Stock price data from Yahoo Finance, news headlines from Reddit WorldNews Channel	Stock price data	Knowledge driven event embedding	Knowledge Driven Temporal Convolutional Network (KDTCN)	Accuracy = 71.8%
Jin, Yang & Liu (2019)	Apple stocks from Yahoo finance, Stock comment dataset from stocktwits	Stock price data	CNN as a base learner for sentiment index	EMD based enhance LSTM(EMD-LSTM) with attention layer	RMSE = 3.196534 MAPE = 1.65 MAE = 2.396121 R^2^ = 0.977388
Li, Wu & Wang (2020)	Hong Kong Exchange daily prices from January 2003 to March 2008, FINET news	Stock price data and Technical indicators	News sentiment analysis using sentiment lexicon	LSTM	Test Accuracy for 3 out of 4 sectors is comparatively better using domain specific dictionary.

## Open issues and research directions

Stock prediction has been an attractive research area for many years. The large amount of textual and numerical data is available to extract significant information and utilize it in prediction techniques. A lot of efficient techniques have been proposed to support data processing and decision making in this area. However, there are a few open problems which are discussed below:

### News preprocessing

Text preprocessing is a non-trivial step before feature selection. There are different techniques that exist for text preprocessing. For instance, removing stop words, lowercasing, stemming, and lemmatizing etc. In (Symeonidis, Effrosynidis & Arampatzis, 2018), comparative experiments are performed on twitter dataset with 16 different preprocessing techniques. This work investigates the significance of these techniques when they are used simultaneously or with different combinations. Twitter dataset contains a significant amount of noise as compared to the text written in a more formal way like news dataset. Preprocessing is also a considerable step for text with less noise in order to reduce feature dimensions and ignore meaningless data. So a thorough comparative evaluation of preprocessing techniques for news dataset will be valuable for the research community, especially dealing with news for stock trend prediction.

### News categorization

In news sensitive stock prediction justified grouping of information plays an important role for efficient information retrieval. For the stock market, news should be properly categorized so that their impact on the stock market can be captured effectively. For instance, news related to politics, terrorism, foreign affairs, finance, economic, and government policies etc., should be categorized accordingly. Furthermore, news related to the stock market should be further categorized into general stock market news, sector related news, and specific stock related news. Supervised techniques are used for news categorization where enough training data exists for acceptable categorization accuracy. But if there is no training data then news is categorized using manual effort. Although unsupervised techniques exist with minimal manual efforts (Usmani & Shamsi, 2020), there is still a need for further efforts required to fully automate these techniques.

### Intensity of news impact

News influences the stock market with different intensities. So there should be a way to estimate the intensity of news impact on stock market. Moreover, it should also be investigated that for how long news has its impact on stock market and how the intensity of news impact gradually decreases over time.

### Impact of bad and good news

On a stock trading day, there can be multiple news with different polarizations that influence market trading simultaneously. For instance, there might be some news that has a positive impact and at the same time some news has a negative impact on the stock market. So estimating their collective effect is also an open research problem in this domain.

### News weighted impact

News from different categories have different influence on the stock market. For instance, regional news has more potential to impact the stock market than global news. In order to gain more insights, news categories should be incorporated and trained separately in the prediction model along with their learned weights.

### Other sources for textual data

Recently a lot of work is being done to capture impact on stock market from news and twitter data. However, integration of additional resources of data like a quarterly or annual reports from a company can be used to further enhance the prediction accuracy. The companies registered in the stock market are required to report at frequent intervals. These reports publish a company activity and financial performance which can help to comprehend future stock trend.

### Hybrid deep learning models

The application of stock prediction by employing Deep Neural Network (DNN) is gaining attention dramatically for many years. CNN and LSTM are examples of deep learning models used for stock prediction (Liang et al., 2019; Yun, Sim & Seok, 2019). However, in Thakkar & Chaudhari (2021) worth of hybrid DNN approaches are discussed which improved prediction accuracy significantly. Vargas, De Lima & Evsukoff (2017) and Liu et al. (2020) proposed hybrid deep learning models by combining the worth of LSTM and CNN. Consequently, there is a need to investigate more sophisticated deep learning approaches by combining basic deep learning models.

## Conclusion

This paper presents an extensive study of stock trend prediction using news and stock prices. It presents a generic approach to implement news sensitive stock prediction model and identifies three main phases. In each phase, challenges are identified and in search of opportunities existing literature is reviewed.

This work has four major contributions. The first contribution of this paper is to provide literature review on this topic. This work elaborates existing research paper and assesses their strengths and limitations. The discussion about existing literature is classified according to different phases in stock prediction namely: (i) forecasting using time series data, (ii) forecasting using financial time series and textual data, (iii) preprocessing and feature extraction in textual and numerical data, (iii) techniques for stock trend prediction using numerical and textual features.

The second contribution is a discussion about key concepts in this scenario that will significantly improve the reader’s comprehension. The third contribution is the identification of challenges and their state of the art solutions in the context of news sensitive stock prediction.

Furthermore, discussion about open issues and future research direction is another important contribution of this survey which is noteworthy for the research community.

## Supplemental Information

10.7717/peerj-cs.490/supp-1 Supplemental Information 1 Artificial neural network.

10.7717/peerj-cs.490/supp-2 Supplemental Information 2 Convolutional neural network.

10.7717/peerj-cs.490/supp-3 Supplemental Information 3 Recurrent neural network.

10.7717/peerj-cs.490/supp-4 Supplemental Information 4 Long short term memory.

10.7717/peerj-cs.490/supp-5 Supplemental Information 5 Summary of stock prediction techniques using financial time series data.

10.7717/peerj-cs.490/supp-6 Supplemental Information 6 Summary of shallow features based text processing techniques.

10.7717/peerj-cs.490/supp-7 Supplemental Information 7 Summary of event based text processing techniques.

## Additional Information and Declarations

### Data Availability

The following information was supplied regarding data availability: This is a survey article, therefore there is no raw data or code.
