# Supplementary figures and images for: News sensitive stock market prediction: literature review and suggestions
Source: PeerJ Comput Sci. 2021 May 4;7:e490. doi: 10.7717/peerj-cs.490 (PMC8114814; doi:10.7717/peerj-cs.490)

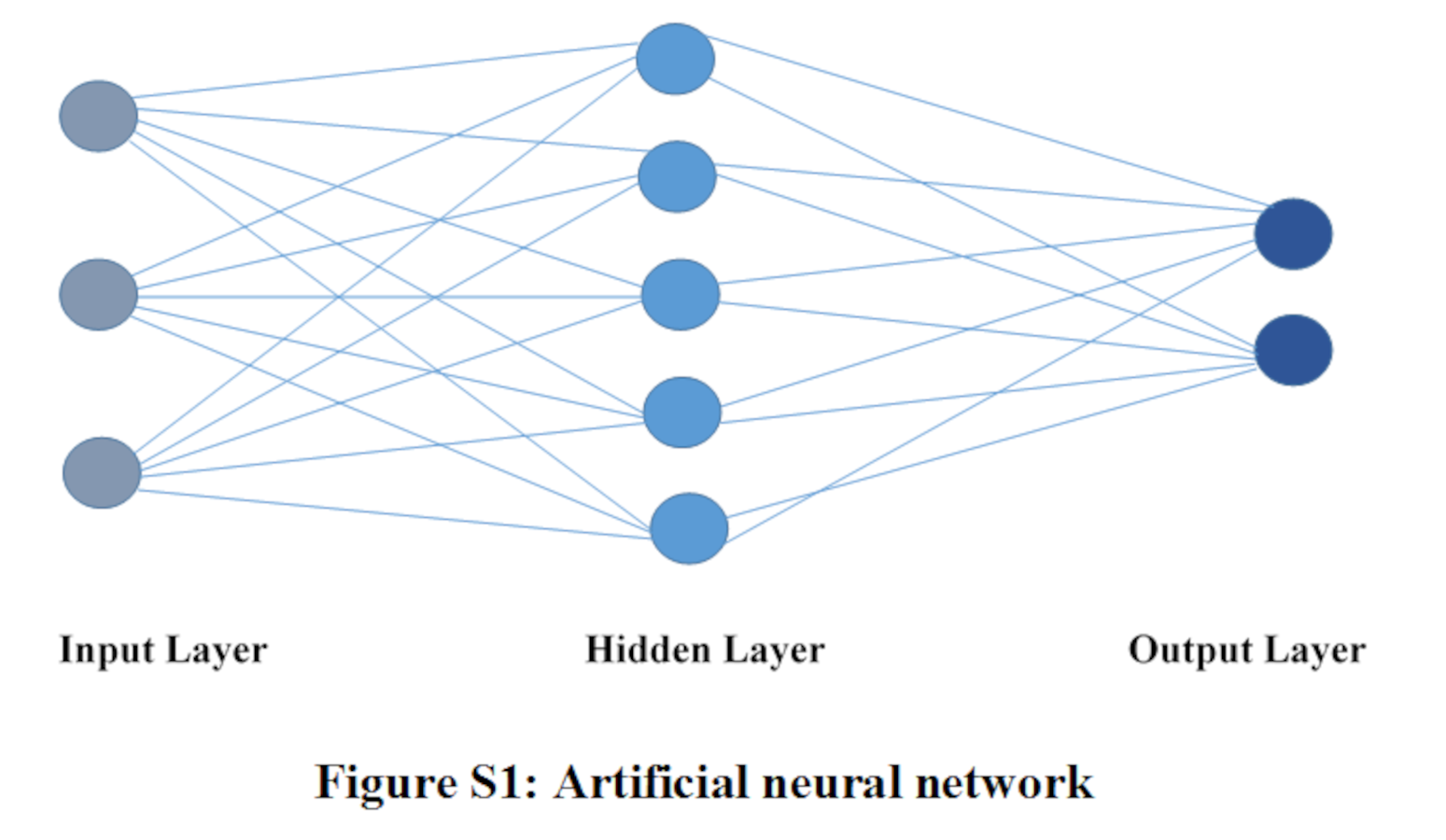

Supplement: Supplemental Information 1 [file peerj-cs-07-490-s001.png]

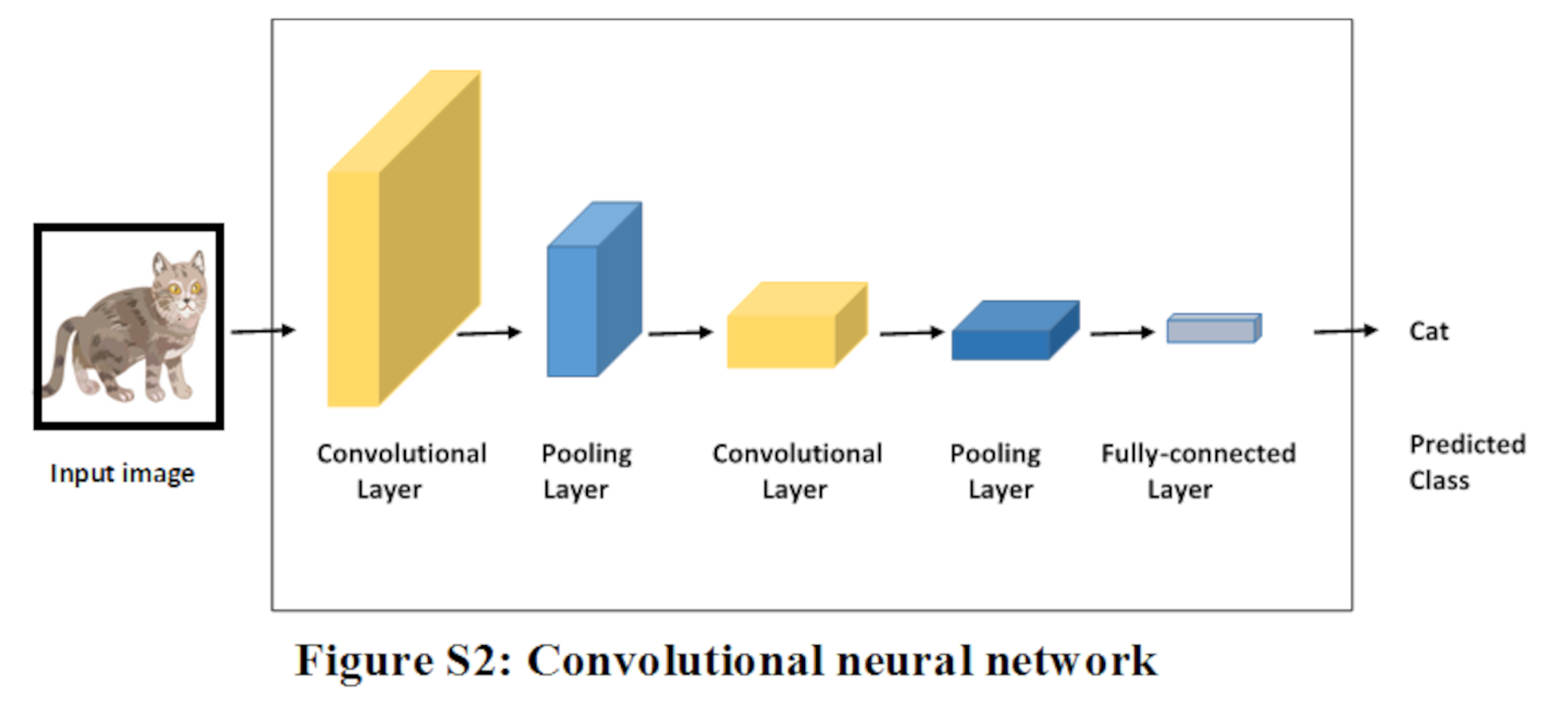

Supplement: Supplemental Information 2 [file peerj-cs-07-490-s002.png]

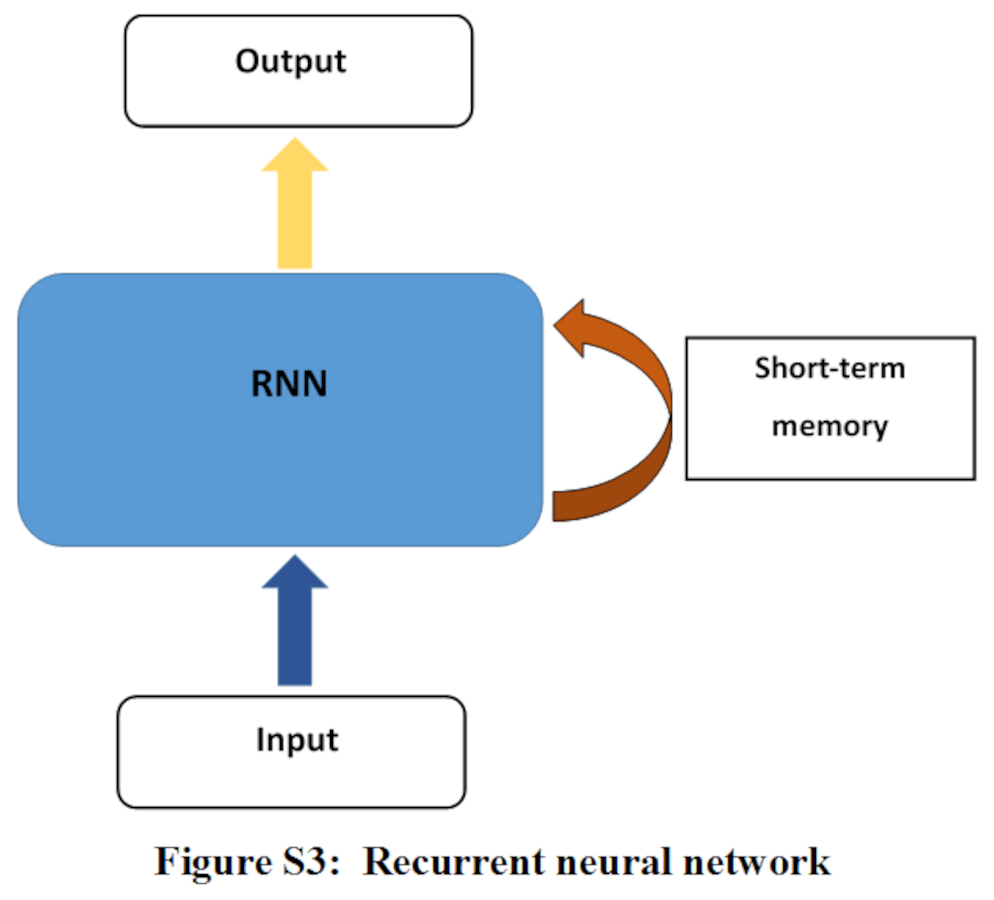

Supplement: Supplemental Information 3 [file peerj-cs-07-490-s003.png]

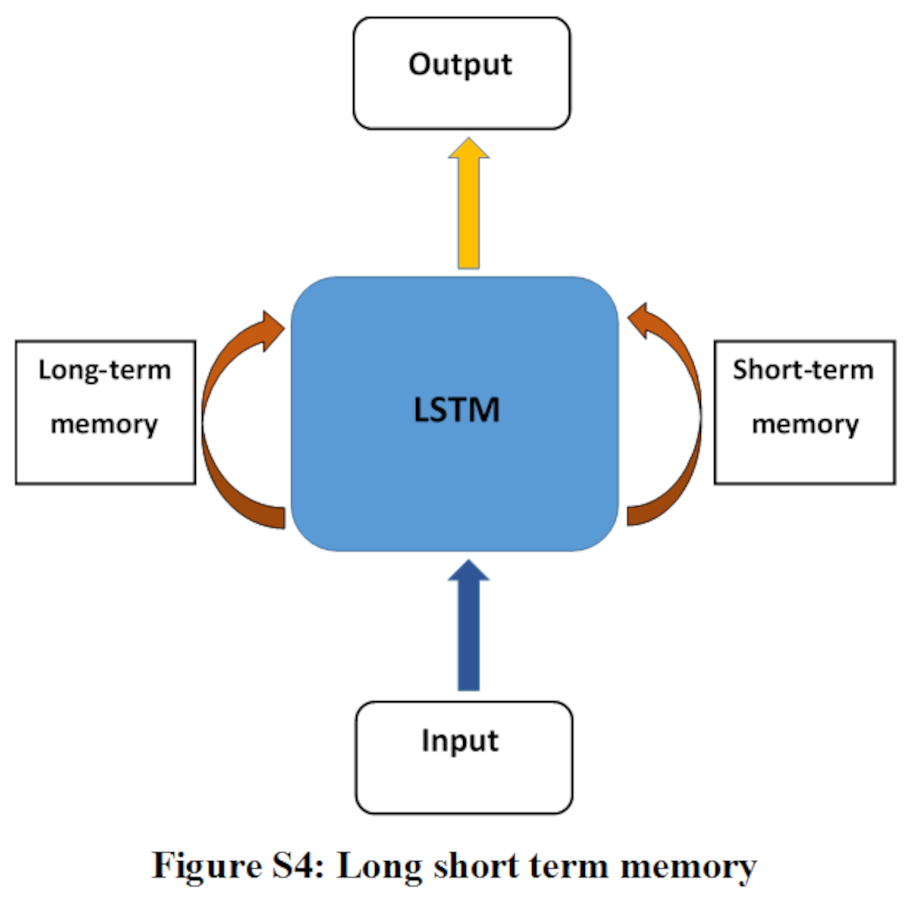

Supplement: Supplemental Information 4 [file peerj-cs-07-490-s004.png]
