# Supplementary material for: News sensitive stock market prediction: literature review and suggestions
Source: PeerJ Comput Sci. 2021 May 4;7:e490. doi: 10.7717/peerj-cs.490 (PMC8114814; doi:10.7717/peerj-cs.490)
Supplement: Supplemental Information 5 [file peerj-cs-07-490-s005.docx]

Table S1: Summary of stock prediction techniques using financial time series data

| **Reference** | **Data Source** | **Prediction Technique** | **Description** | **Performance Metric** |
| --- | --- | --- | --- | --- |
| (Huang, Nakamori et al. 2005) | NIKKEI 225 index From Yahoo | SVM, QDA, LDA, and EBNN | SVM performed better than others. | Hit ratio |
| (Ou and Wang 2009) | Hang Seng index From Yahoo Finance | LDA, QDA, k-NN, NB, NN, Logit model, SVM, LS-SVM, Tree based and Bayesian classification | SVM and LS-SVM performed better than other techniques used in experiments. | Hit rate and Error rate |
| (Kara, Boyacioglu et al. 2011) | Istanbul Stock Exchange (ISE) | ANN, SVM | ANN produced better accuracy than SVM | T-test |
| (Sun and Li 2012) | China Stock Market and Accounting Research Database | SVM ensemble | SVM ensemble is better than SVM | T-test |
| (Huang, Huang et al. 2012) | Nasdaq composite index | SVM based on wavelet  kernel function | Proposed techniques demonstrated better results than Gaussian kernel SVM and polynomial kernel SVM | MSE, MAE |
| (Liu and Wang 2012) | Market indexes SAI,  SBI, DJI and IXIC | LNN based on random time strength function | Random time strength function is analyzed using volatility and drift parameters. | MAPE |
| (Ticknor 2013) | Microsoft Corp. and Goldman Sachs  Group Inc. stock. | BNN | Proposed model showed better results than fusion model and ARIMA model. | MAPE |
| (Shahpazov, Velev et al. 2013) | Bulgarian Stock exchange | MLP,RBF, GRNN | Different neural networks were analyzed. GRNN performed better. | RMSE |
| (Cheng and Wei 2014) | Taiwan stock exchange | SVR based on EMD | Comparative analysis with SVR model and autoregressive (AR) model demonstrates proposed technique superiority. | RMSE |
| (Wang and Wang 2015) | Shanghai Stock Exchange, Dow Jones Industrial Average, Hong Kong Hang Seng 300 Index (HS300), and Standard & Poor’s (S&P500) | STNN with PCA | PCA-STNN showed better results than BPNN PCA-BPNN and STNN. | MAE, RMSE, and  MAPE |
| (Mann and Kutz 2016) | Chinese Stock Market | DMD to investigate market profitability | DMD performed well for sideways market | SPA test |
| (Kuttichira, Gopalakrishnan et al. 2017) | National Stock Exchange | DMD for price forecasting | DMD results were better than ARIMA results | MAPE |
| (Nelson, Pereira et al. 2017) | IBovespa index from the BM&F Bovespa stock exchange | LSTM | Multi-Layer Perceptron, Random Forest, and a pseudo-random  Model are used as baseline model | Accuracy |
| (Arratia and Sepúlveda 2019) | S&P 500 | CNN | Numeric time series data is converted to images and pass as input to CNN model for improved classification accuracy | Accuracy |
| (Yujun, Yimei et al. 2020) | S&P 500  Hang Seng Index  Australian Securities Exchange  Frankfurt Stock Exchange | LSTM with EMD  and  LSTM with EEMD | Proposed hybrid approaches were compared with other regression methods and demonstrated is superiority. | RMSE, MAE, R^2^ |

^Abbreviations: MSE, Mean Square; MAE, Mean Absolute Error; RMSE, Root Mean Squared Error; MAPE, Mean Absolute Percentage Error; R2, R- Squared^
