# Supplementary material for: News sensitive stock market prediction: literature review and suggestions
Source: PeerJ Comput Sci. 2021 May 4;7:e490. doi: 10.7717/peerj-cs.490 (PMC8114814; doi:10.7717/peerj-cs.490)
Supplement: Supplemental Information 6 [file peerj-cs-07-490-s006.docx]

Table S2: Summary of shallow features based text processing techniques

| **Reference** | **Data Set (Textual)** | **Feature Type** | **Prediction Technique** | **Performance Metric** |
| --- | --- | --- | --- | --- |
| (Cho, Wuthrich et al. 1999) | Financial News | BoW | Probabilistic rules | Accuracy |
| (Zhai, Hsu et al. 2007) | Financial News | BoW | SVM | Accuracy |
| (Schumaker and Chen 2009) | Financial News | BoW, noun phrases, and named entities | SVM | MSE |
| (Groth and Muntermann 2011) | Announcements | BoW | NB, KNN, NN, and SVM | t-values |
| (Hagenau, Liebmann et al. 2013) | Financial News | BoW, Noun Phrases, Word combinations, and N-grams | SVM | Precision, Recall, and F1-measure |
| (Luss and d’Aspremont 2015) | Financial News | BoW, | MKL | Accuracy, Sharp ratio |
| (Dadgar, Araghi et al. 2016) | General News | BoW | SVM | Precision, Recall, and F-measure |
| (Li, Xie et al. 2016) | Financial News | BoW | ELM | p-value |
| (Vargas, De Lima et al. 2017) | Financial News | Word embedding, Sentence embedding | CNN-LSTM | Accuracy |
| (Garcia-Lopez, Batyrshin et al. 2018) | Tweets | Bow, Word embedding | Classifiers in scikit-learn API , Majority Vote, Voting Classifier | Accuracy |
| (Yun, Sim et al. 2019) | Title of Newspaper Articles | Word embedding | CNN | Accuracy |

^Abbreviations: MSE, Mean Square Error; MAE, Mean Absolute Error; RMSE, Root Mean Squared Error; MAPE, Mean Absolute Percentage Error^
