# Supplementary material for: News sensitive stock market prediction: literature review and suggestions
Source: PeerJ Comput Sci. 2021 May 4;7:e490. doi: 10.7717/peerj-cs.490 (PMC8114814; doi:10.7717/peerj-cs.490)
Supplement: Supplemental Information 7 [file peerj-cs-07-490-s007.docx]

Table S3: Summary of event based text processing techniques

| **Reference** | **Data** | **Data Source** | **Class** | **Domain** | **Description** |
| --- | --- | --- | --- | --- | --- |
| (Naughton, Kushmerick et al. 2006) | Electronic News | News stories related to Iraq war are collected from 46 different sources | Data | War | War related event extraction, WEKA tool used for classification. |
| (Tanev, Piskorski et al. 2008) | Electronic News | Europe Media Monitor- a web based news cluster system | Data | Security | Violent and natural disaster related event extraction. |
| (Jungermann and Morik 2008) | Document | Public collection of minutes of Germen parliament's plenary sessions and petitions. | Hybrid | Politics | RapidMiner as an information extraction tool. |
| (Borsje, Hogenboom et al. 2010) | Electronic News | RSS News Feeds from MarketWatch, Yahoo!, and Reuters | Knowledge | Finance | Lexico-semantic pattern based event extraction |
| (Björne, Ginter et al. 2010) | Article | PubMed | Hybrid | Biomedical | PubMed driven knowledge base |
| (IJntema, Sangers et al. 2012) | Electronic News | RSS News Feeds from Yahoo! Business and Technology | Knowledge | Finance and Politics | Lexico-Semantic based pattern are better than lexico-syntactic based patterns |
| (Hogenboom, Hogenboom et al. 2013) | Electronic News | Reuters Business and Technology News and The New York Times Business News | Knowledge | Finance | semantic lexicons and ontologies based event extraction |
| (Nuij, Milea et al. 2014) | Electronic News | Reuters | Knowledge | Finance | Ontologies based event extraction using ViewerPro tool |
| (Ding, Zhang et al. 2014) | Electronic News | Reuters and Bloomberg | Data | Finance | Financial event extraction using OpenIE technology |
| (Ding, Zhang et al. 2015) | Electronic News | Reuters and Bloomberg | Data | Finance | Financial event extraction using OpenIE technology and event embedding |
| (Ding, Zhang et al. 2016) | Electronic News | Reuters and Bloomberg | Data | Finance | Financial event extraction using OpenIE technology and knowledge driven event embedding |
| (Chen, Zou et al. 2019) | Electronic News | Reuters | Knowledge | Finance | Domain expert based finance event dictionary |
| (Deng, Zhang et al. 2019) | Electronic News | Reddit WorldNews | Knowledge | Finance | Structured events embedding using knowledge graph |
